# Near-Net Forming Complex Shaped Zr-Based Bulk Metallic Glasses by High Pressure Die Casting

**DOI:** 10.3390/ma11112338

**Published:** 2018-11-21

**Authors:** Lehua Liu, Tao Zhang, Zhiyuan Liu, Chunyan Yu, Xixi Dong, Liangju He, Kuan Gao, Xuguang Zhu, Wenhao Li, Chengyong Wang, Peijie Li, Laichang Zhang, Lugee Li

**Affiliations:** 1 State Key Laboratory of Tribology, Department of Mechanical Engineering, Tsinghua University, Beijing 100084, China; lh-li14@mails.tsinghua.edu.cn (L.L.); Xixi.Dong@brunel.ac.uk (X.D.); helj@mail.tsinghua.edu.cn (L.H.); 2 Institute of Manufacturing Technology, Guangdong University of Technology, Guangzhou 510000, China; cywang@gdut.edu.cn; 3 Guangdong Provincial Key Laboratory of Micro/Nano Optomechatronics Engineering, College of Mechatronics and Control Engineering, Shenzhen University, Shenzhen 518060, China; zyliu@szu.edu.cn; 4 College of Physics and Energy, Shenzhen University, Shenzhen 518060, China; 5 Institute of Eontech New Materials Co., Ltd., Dongguan 523662, China; gaokuan6@163.com (K.G.); zhuxuguang@yihaometal.com (X.Z.); vino_01@163.com (W.L.); lli@lli.cc (L.L.); 6 School of Engineering, Edith Cowan University, 270 Joondalup Drive, Joondalup, WA 6027, Australia; lczhangimr@gmail.com

**Keywords:** bulk metallic glass, industrialized application, high pressure die casting

## Abstract

Forming complex geometries using the casting process is a big challenge for bulk metallic glasses (BMGs), because of a lack of time of the window for shaping under the required high cooling rate. In this work, we open an approach named the “entire process vacuum high pressure die casting” (EPV-HPDC), which delivers the ability to fill die with molten metal in milliseconds, and create solidification under high pressure. Based on this process, various Zr-based BMGs were prepared by using industrial grade raw material. The results indicate that the EPV-HPDC process is feasible to produce a glassy structure for most Zr-based BMGs, with a size of 3 mm × 10 mm and with a high strength. In addition, it has been found that EPV-HPDC process allows complex industrial BMG parts, some of which are hard to be formed by any other metal processes, to be net shaped precisely. The BMG components prepared by the EVP-HPDC process possess the advantages of dimensional accuracy, efficiency, and cost compared with the ones formed by other methods. The EVP-HPDC process paves the way for the large-scale application of BMGs.

## 1. Introduction

Bulk metallic glass (BMG) is always regarded as one of the most promising materials because of its ultrahigh strength, large elasticity, and excellent corrosion resistance, and thus can be applied in wide range of fields [1,2]. However, a long-standing problem for this type of material is that it is difficult to mold into complex shapes using the casting method. To overcome this bottleneck, several alternative approaches, such as thermoplastic forming (TPF) [3,4] and additive manufacturing (AM) process [5], were developed. Through these approaches, the BMG parts with complex geometric shapes have been produced successfully [3,4,5]. However, some inherent problems, including crystallization, cracking, relatively low efficiency (in AM process) [5,6], extremely narrow time window for shaping, and difficulty in forming a hollow structure (in the TPF process) [7], were not well solved, impeding the application of these methods in the mass production of BMGs. In order to achieve the industrial production of BMG parts, some other methods, including low pressure die casting [8] and squeeze casting [9], have also been reported in the past decade. However, up until now, only low fusion point Mg-based [8] and Ca-based [10] BMG parts with simple geometries like rods or plates have been fabricated successfully by these approaches. Currently, the main technology available for the large-scale forming of BMG parts with a complex shape is the injection molding process developed by Liquidmetal Technologies, Inc. [11]. In this approach, the mother alloy is melted by induction heating in a horizontal cold-crucible, and then molten metal is confined to a specific area by a magnetic field. When the metal is completely melted, the molten metal is injected into the mold using a plunger rod, forming a BMG part. However, there is an Achilles heel in this approach, that is, the non-uniformity of the temperature field during heating, mainly caused by the horizontal layout of the cold-crucible. The non-uniformity of the heating would result in the sometimes-incomplete melting of the master alloy, thereby weakening the glass forming ability [12] and inducing the instability of the product quality [13,14]. Besides, as a commercialized technology, the injection molding of a BMG route is very confidential, and few results have been reported in an academic paper, which is disadvantageous to the prosperity of the related disciplines. Therefore, exploring a new but more general approach to achieve the large-scale production of BMG parts becomes increasingly urgent and necessary.

High pressure die casting (HPDC) is a metal forming process that is widely used in real industry for forming aluminum, magnesium, zinc, and copper alloy components [15]. It possesses several advantages, such as a high cooling rate, high productivity, and the ability to form near net shapes. Therefore, the HPDC process is also considered as the one of the methods with the most potential to be applied to the large-scale production of BMG components with sophisticated geometries. Recently, low oxygen affinity Fe-based bulk metallic glass with a key-shape was produced successfully using a traditional HPDC method in an open atmosphere [16]. However, most glass formers are extremely oxidizable, and thus are difficult to be synthesized directly by this route. In order to form highly oxygen-sensitive BMGs, Inoue et al. proposed a vertical vacuum HPDC solution, as shown in the literature [17,18]. In this approach, the melting of mother alloy was conducted in a sealed sleeve manufactured by steel. However, up until now, only low melting point Mg-based [18] and La-based alloys [17] were prepared successfully using this approach. With regard to the most common Zr-based BMGs, especially for Be-free glass formers, its forming by high pressure die casting still encounters challenges because of the limitations of the molding methods. Therefore, developing a new HPDC route enabling the formation of BMGs with a higher melting point and chemical activity would have huge engineering and academic significance.

In this study, a novel HPDC method, named the entire process vacuum high pressure die casting (EPV-HPDC), was developed by modifying just the existing traditional horizontal HPDC equipment. Various Zr-based BMG plates with a critical thickness of 3 mm were successfully prepared using the EPV-HPDC and industrial grade purity raw materials. Meanwhile, several industrial BMG parts with complex shapes that are hard to form using the other forming processes were molded precisely using the EPV-HPDC process. The results confirm that the EPV-HPDC process is feasible for casting high-melting-point BMG parts with a higher chemical activity. We anticipate that the newly developed EPV-HPDC process will facilitate the industrial production and large-scale application of Be-free Zr-based BMGs.

## 2. Experimental

### 2.1. Entire Process Vacuum High Pressure Die Casting (EPV-HPDC) Equipment

**Figure 1:**
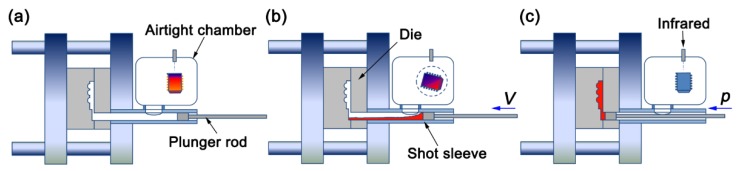
Illustration of the EPV-HPDC equipment and operating mode: (**a**) loading materials and melting, (**b**) pouring liquid metal into the shot sleeve and filling die, and (**c**) solidifying under pressure.

The EPV-HPDC equipment was modified based on a standard horizontal cold chamber HPDC machine (Eontec Co., Ltd, Dongguan, China) with a mold clamping force of 280 T. The schematic diagram of the developed EPV-HPDC is presented in Figure 1. The entire casting processes, including the melting, pouring, filling die, and solidification, are integrated into an airtight chamber, which ensures the formation of the components in an extreme vacuum environment. The sleeve, plunger, and mold here were made from heat resistant steel (H13). Unlike the vertical HPDC solution proposed by Inoue et al. [17,18], in the present horizontal EPV-HPDC, the melting of the mother alloy is performed in an oxide crucible, which can avoid heating the metal by a sleeve and can improve the operating temperature range of the equipment. The highest smelting temperature in the present EPV-HPDC is 1773 K, and the highest casting temperature is 1533 K, which enables the formation of most of the glass formers. The temperature of the molten metal is detected and controlled using an infrared sensor. It should be emphasized here that the EPV-HPDC process is different from the injection of a molding solution of BMGs [11], as well as from the traditional vacuum assisted HPDC [19]. The layout of the vertical and hot crucible in the present EVP-HPDC can guarantee a more uniform and controllable temperature field during heating, compared with that in the solution of horizontal and cold crucible proposed by Liquidmetal Technologies, Inc. (Rancho Santa Margarita, CA, USA) [11]. With regard to the traditional vacuum assisted HPDC, it cannot be used to prepare the materials that are easily oxidized, because the vacuumizing in this process is triggered only when the molten metal is about to be filled in a mold cavity [19].

### 2.2. EPV-HPDC Experiment

Several metallic glass formers with a composition (at.%) of Zr_55_Cu_30_Ni_5_Al_10_ (Zr55), Zr_52.5_Ti_5_Cu_17.9_Ni_14.6_Al_10_ (Vit105), Zr_57_Nb_5_Cu_15.4_Ni_12.6_Al_10_ (Vit106), and Zr_46.5_Cu_47.5_Al_4_Co_1_Sn_1_ (ZrCu-based, a marginal bulk glass former) were used in the present study to present the forming ability of the EPV-HPDC method. The master alloys were prepared in 5 kg batches, by induction melting the mixtures of the respective industrial grade purity elements (purity of zirconium 99.40%, and the others were 99.99%) and 0.20 at.% rare earth yttrium in a high purity (99.99%) argon atmosphere, and then were broken into granular feedstock with a size smaller than 30 mm in a general crusher. BMG plates with different thicknesses (cross-sectional areas of 1 mm × 10 mm, 2 mm × 10 mm, and 3 mm × 10 mm) were cast directly in a multi-cavity mold using the EPV-HPDC method. The detail EPV-HPDC process involved several steps. Firstly, the granular feedstock was loaded into the airtight chamber and then melted using an induction heating system with the vacuum pressure of 10 Pa (Figure 1a). Once the alloy was fully viscous, it was poured into the shot sleeve with casting temperatures of 1233 K for Zr55, Vit105, and Vit106, and 1313 K for the ZrCu-based alloy, and then forced into the mold using a plunger with a velocity of 1.1 m/s (Figure 1b). After filling the die, the molten metal was frozen in the mold under pressure (~40 MPa), forming a net-shape part (Figure. 1c). A die lubricant was not used in the EPV-HPDC process. The casting process of the EPV-HPDC in the form of a video is displayed in the Supplementary Materials.

### 2.3. Microstructural and Performance Characterization

The structures of the as-cast samples were measured using X-ray diffraction with Cu Kα radiation (XRD, Brucker Advance D8, Bruker AXS, Billerica, Germany). The thermal stability and crystallization behavior were characterized using differential scanning calorimetry (DSC, Parkin-Elmer DSC8000, Waltham, MA, USA) under a purified argon atmosphere with a heating rate of 20 K/min. The surface roughness was measured by profilometer (KLA tencor D-300, KLA-Tencor Corporation, Milpitas, CA, USA). The morphologies of the casting surfaces were observed using a laser scanning confocal microscope (KEYENCE, VK-X250, Keyence Corporation, Osaka, Japan). The porosity distribution of the as-cast samples was detected by using an X-ray fluoroscopy (YXLon, Y. Couger, YXLON, Hamburg, Germany) and a three-dimensional X-ray computer tomography (CT) machine (Diondo, d2, diondo GmbH, Hattingen, Germany). The compression tests were conducted on a mechanical testing system with a strain rate of 5 × 10^−4^ s^−1^. The compressive samples with an aspect ratio 2:1 were removed from the intermediate region of the plates with thickness of 1 mm. The fracture surface was observed using a ZEISS SUPRA 55 Field Emission Scanning Electron Microscope (SEM, Carl Zeiss Microscopy GmbH, Jena, Germany).

## 3. Results and Discussion

**Figure 2:**
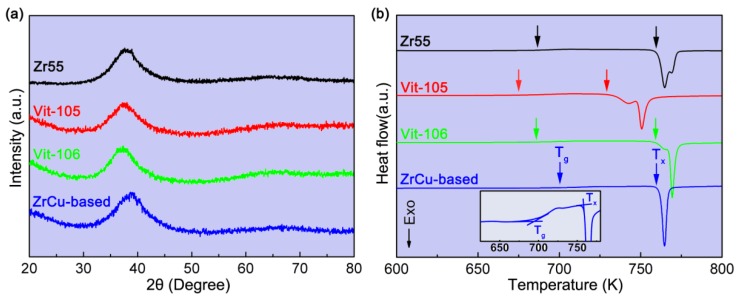
(**a**) XRD patterns of the Zr_55_Cu_30_Ni_5_Al_10_ (Zr55), Zr_52.5_Ti_5_Cu_17.9_Ni_14.6_Al_10_ (Vit105), Zr_57_Nb_5_Cu_15.4_Ni_12.6_Al_10_ (Vit106), and Zr_46.5_Cu_47.5_Al_4_Co_1_Sn_1_ (ZrCu-based) samples with different thicknesses; (**b**) DSC curves for the different BMGs fabricated using EPV-HPDC, the glass transition temperature (*T*_g_) and the crystallization temperature (*T*_x_) are arrowed. The inset in (**b**) displays the details of the glass transition behavior of the ZrCu-based BMG.

Figure 2 displays the XRD and DSC patterns of the BMGs prepared using EPV-HPDC. Figure 2a shows that all of the samples have a board diffraction peak, and no diffraction peak corresponding crystalline peak is seen, suggesting that an amorphous structure was produced successfully in the Zr55, Vit105, and Vit106 specimens, with cross-sectional area of 3 mm × 10 mm, as well as in the ZrCu-based alloy with a cross sectional area of 1 mm × 10 mm by using industrial grade purity raw materials (Figure 2a). The DSC analysis shows that all of the specimens have a distinct glass transition followed by a crystallization process (Figure 2b). Glass transition temperature, *T*_g_, is 676 K, 654 K, 670 K, and 699 K, and the crystallization temperature, *T*_g_, is 760.8 K, 730 K, 726 K, and 759 K for Zr55, Vit105, Vit106, and ZrCu-based BMGs, respectively. The enthalpies of crystallization calculated by the areas of the crystallization peak are 45.2 ± 2.5 J/g, 43.7 ± 2.8 J/g, 46.4 ± 1.8 J/g, and 53.76 ± 2.1 J/g for the Zr55, Vit105, Vit106, and ZrCu-based BMGs, respectively. These values are equal to the corresponding ones fabricated using copper mold suction-casting in the laboratory, confirming that fully glassy structures have been obtained using the EPV-HPDC process, even in the ZrCu-based BMG with a marginal glass-forming ability. Although the critical sizes of the BMGs prepared by the EPV-HPDC process here are smaller than the reported values of the counterparts fabricated by high purity material in laboratory [20,21], they are comparable to the ones prepared from the industrial purified raw materials. For instance, a previous study showed that a 6 mm in diameter of rod was the critical size of the Zr55 BMG fabricated from industrial purified raw materials [22]. In this study, the EPV-HPDC process was capable of forming amorphous plates with a size of 3 mm × 10 mm, of which the equivalent diameter, determined by $\sqrt{4ab/\pi }$, is about 6.1 mm, where *a* and *b* are the length and width of the plate, respectively. The results indicate the glass forming ability of EVP-HPDC without drastically declining, compared with that of suction casting [22], even though the mold temperature in the present process (533 K) is far higher than that in laboratory (293 K). The relative high glass forming ability in the EVP-HPDC process is mainly ascribed to the established larger heat transmission coefficient, resulting from a better thermal contact between the mold and molten metal under pressure [23].

In the past few years, even though various Mg-, La- and Fe-based BMGs have been fabricated successfully by high pressure die casting process [17], the preparation of the Zr-based BMG by HPDC is still difficult and is rarely reported, because of the relatively high melting point and oxygen affinity. In this work, the success in forming Be-free Zr-based BMGs, especially the ZrCu-based glass former, confirms that the EPV-HPDC process is feasible to fabricate Zr-based BMGs for applications.

**Figure 3:**
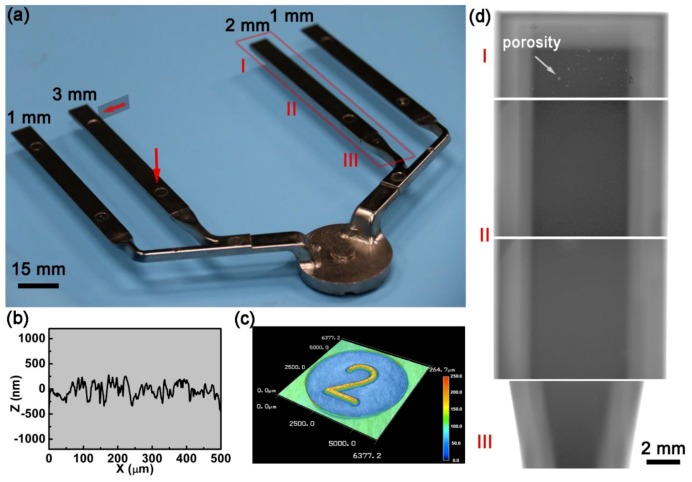
(**a**) Image of BMG sample (Zr_55_Cu_30_Ni_5_Al_10_, Zr55) fabricated by the EPV-HPDC process. The red arrow indicates from where the samples were taken for X-ray diffraction, and the DSC measurements. All of the breadths of the plates are 10 mm; (**b**) Profilogram of surface of casting; (**c**) detail of a miniature embossing character in the surface of casting; (**d**) X-ray images from the representative regions of casting in (**a**).

Figure 3a displays an image of the typical Zr55 castings (including runner system). It is clearly seen that the BMG part displays a good surface luster (Figure 3a). Figure 3b displays the local contour measured by a profilometer. The roughness shows that the Zr55 BMG casting has a low *R*_a_ value (~0.26 μm), which is the same as that of the die cavity (Figure 3b). By comparing the design sizes, it is found that the dimensional deviation of the various thicknesses of the BMG plates is within ± 0.25%, of which the magnitude is usually common for the productions manufactured by computer numerical control (CNC) machining. The high dimensional accuracy and low surface roughness are mainly attributed to the characteristics of the EPV-HPDC method, as well as the solidification nature of the glass forming liquid. On the one hand, the filling and solidification of EPV-HPDC always proceed under high pressure. As such, the molten metal has the capacity to precisely pattern the complex mold cavity. On the other hand, the molten metal during cooling is able to maintain the as-filled morphology with little shrinkage, because of the lack of phase transformation for glass forming liquid during the vitrification process [4]. 

Furthermore, it is found that the EPV-HPDC process can cast even the fine surface feature, such as the character “2”, with minimum thickness of 250 μm (Figure 3c). Previous studies reported that the minimum molding section thickness of HPDC for traditional materials, such as aluminium alloys, was about 2–2.5 mm [24]. The ability to form parts with a thickness of 0.25 mm here indicates that the present EPV-HPDC process has a huge advantage in casting BMG components with a fine construction. The high forming ability here is ascribed to the character of the high mold-filling capacity of the glass former under a low temperature. With regard to a traditional casting alloy, such as an Al-Si alloy, the filling must be finished before the solidification. Otherwise, the primarily quenched solid would obstruct the runner of the casting system, leading to the abortion of the shaping of complex structural especially thin-walled parts. Therefore, the available temperature window for shaping should be within the casting temperature, *T*_c_, and solidus temperature, *T*_s_ [25]. For the glass former, however, the solidus temperature, *T*_s_, can be as low as *T*_g_ (i.e., ~680 K for Zr55). The available temperature window for shaping is about 557 K, which is several times larger than that of the traditional materials (~150 K), such as an Al-Si alloy. Therefore, the mold-filling capacity of the bulk metallic glass in the HPDC process is far larger than that of the traditional casting alloy, and thus has the ability to form a fine structure with a small thickness.

Furthermore, the porosity distribution in the casting was detected using an X-ray fluoroscopy, as shown in Figure 3d. Most of the regions display a very uniform structure. No obvious gas pores were observed in the samples, except for the head region. The relative densities of the BMG samples fabricated using the EPV-HPDC process are measured to be above 0.98, which is much higher than the values reported previously in the BMGs (about 0.8) fabricated by the developed die casting process [17,18], as well as higher than that of the aluminum and magnesium alloy components formed by the traditional HPDC process [26]. The relative high density is mainly ascribed to using the optimized process parameters and high vacuum degree during the entire casting process (~10 Pa), which is far higher than that in the vacuum assisted HPDC (~5 × 10^3^ Pa) [21].

**Figure 4:**
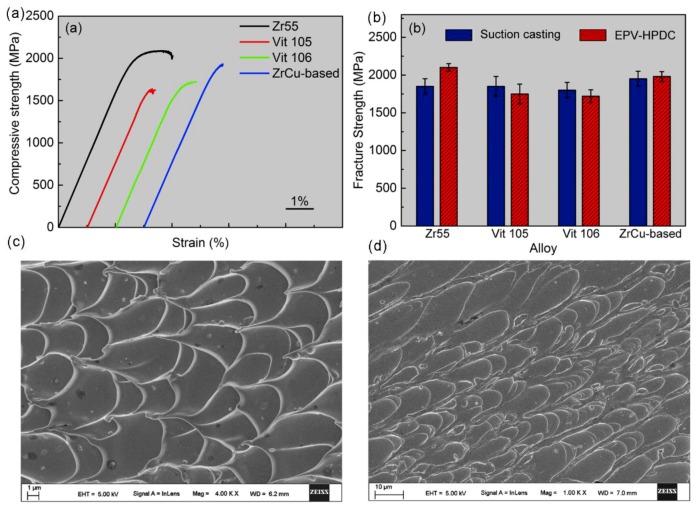
(**a**) Engineering stress–strain curves for the Zr_55_Cu_30_Ni_5_Al_10_ (Zr55), Zr_52.5_Ti_5_Cu_17.9_ Ni_14.6_Al_10_ (Vit105), Zr_57_Nb_5_Cu_15.4_Ni_12.6_Al_10_ (Vit106), and Zr_46.5_Cu_47.5_Al_4_Co_1_Sn_1_ (ZrCu-based) BMG samples, respectively; (**b**) comparison of the fracture strengths of BMGs prepared by suction casting and the EPV-HPDC process by using industrial grade purity elements; (**c**) SEM of the fracture morphologies of the sample Vit106; (**d**) SEM of fracture morphologies of the sample Vit105.

Figure 4a displays the engineering stress–strain curves of the BMGs cast using the EPV-HPDC process. The fully glassy structural Zr55 BMG displays an ultrahigh strength of 2105 ± 50 MPa, and a plastic strain of 1.8 ± 1.4% (Figure 4a). Less plastic strains are detected in the other samples. The fracture strengths for the Vit105, Vit106, and ZrCu-based BMGs are measured to be 1750 ± 130 MPa, 1720 ± 84 MPa, and 1950 ± 100 MPa, respectively. These values are comparable to those of the samples cast in the laboratory (Figure 4b), further confirming that the porosities of the castings are low. But the plasticity for all of the samples is far lower than that of the castings fabricated by copper mold suction casting. In the EVP-HPDC process, the molten metal solidifies under a relatively lower cooling rate and higher solidification pressure compared with the suction casting in the laboratory. The lower cooling rate provided more time for the supercooling liquid to relax to an “ideal state” possessing less free volume [27,28]. Furthermore, the high solidification pressure also facilitates the forming of a denser structure [12]. Studies indicate that the decreasing free volume in the as cast BMGs results in the formation of less shear bands during deformation, thereby causing a decrease of plasticity [28,29]. Therefore, the BMGs fabricated by EPV-HPDC display a lower plasticity. Figure 4c,d displays the fracture morphologies of the sample Vit106 and Vit105 fabricated using the EPV-HPDC process. The fractography displays abundant peak-to-peak dimple patterns, which are typical characteristic of BMGs. The size of the dimple-like structures on the fracture surface, which can be defined as average spacing between ridges of dimples surrounding the center of each dimple zone, is ~5 μm and ~7 μm for Vit106 and Vit105, respectively. These values are close to the reported value of the Vitreloy alloy [30]. Moreover, very few pores were observed in the fractography, which is consistent with the X-ray images (Figure 3d).

**Figure 5:**
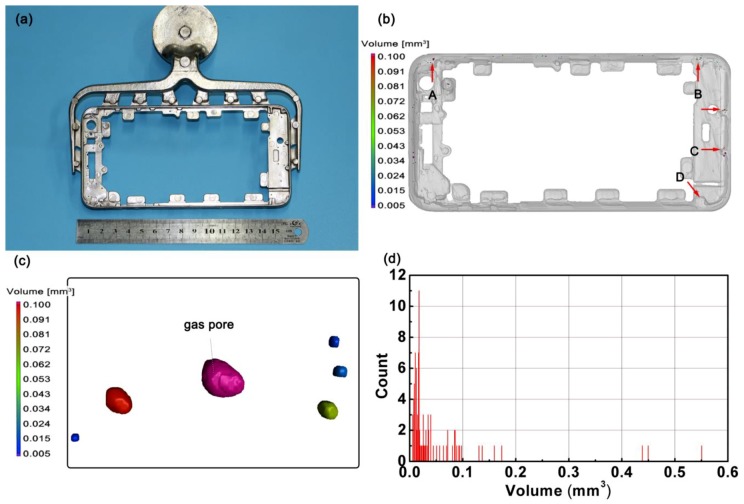
(**a**) Image of Zr_55_Cu_30_Ni_5_Al_10_ BMGs smartphone frame and the corresponding runner system; (**b**) the 3D distribution of the porosity in the smartphone frame, A, B, C, and D denote different regions; (**c**) morphologies of the gas pores; (**d**) statistics of the pores with a different size in casting.

As the filling time of the molten metal into die can be completed within 50 ms in the EVP-HPDC system, and the critical cooling rate for most of the BMG formers is at a magnitude of 10^2^ K/s [31], theoretically, any BMGs can be cast into the sophisticated geometries using EPV-HPDC process to break through the limitation of the trade-off between the cooling rate and the available time window of shaping. Herein, the EPV-HPDC is applied to produce a real smartphone frame in order to test its ability to form the complex shape. Figure 5a displays an as-cast smartphone frame and corresponding EPV-HPDC gating system. The boundary dimension of the smartphone is about 155 mm × 85 mm × 10 mm, and the minimum thickness is about 0.5 mm. It has been found that the component is formed successfully and displays a good surface smoothness (Figure 5a). A three dimensional X-ray computed tomography (CT) is employed to detect the defects in the part. The distribution of the porosity is displayed in Figure 5b. It is found that the porosity is mainly distributed in some bulges of the part, as indicated by the red arrows (Figure 5b). The morphologies of the pores are displayed in Figure 5c. It shows that most of the gas pores are globular. The statistics of the pore sizes are exhibited in Figure 5d. The existed pores mostly have a volume of less 0.1 mm^3^. By summing the volumes of the pores, the porosity of the part is estimated to be ~0.9%, which is much lower than the values reported previously in Mg-based [18], Ca-based [8], and La-based [17] BMGs (porosity > 10%) fabricated using the developed die casting process. 

**Figure 6:**
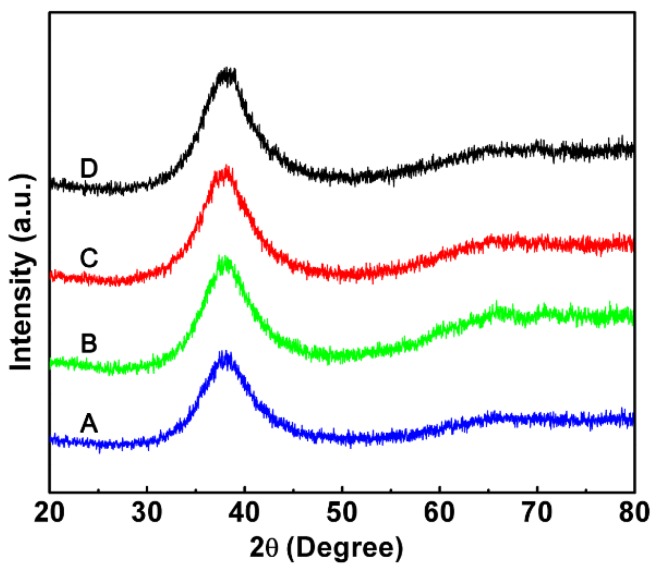
XRD patterns measured from the A, B, C, and D region of the smartphone frame in Figure 5b.

Afterwards, the structure of the smartphone frame was characterized by XRD. Although there are several pores in the part, the XRD tests from the thickest regions, indicated by the arrows, show that the as-cast part possesses a completely glassy structure (Figure 6). Previous reports show that gas pores in casting is an efficient heat-insulating medium that retards heat transfer in the melt, compared with the regions without porosity, leading to a lower local solidification rate [26], which can thus induce crystallization. The results indicate that the small number of pores present in the Zr55 BMG have a negligible impact on the glass forming ability. The success in forming the smartphone frame here demonstrates that the EPV-HPDC process is feasible to a near-net shape BMG part with sophisticated geometries, and paves the way for the Zr-based BMGs to be applied in wider fields.

**Figure 7:**
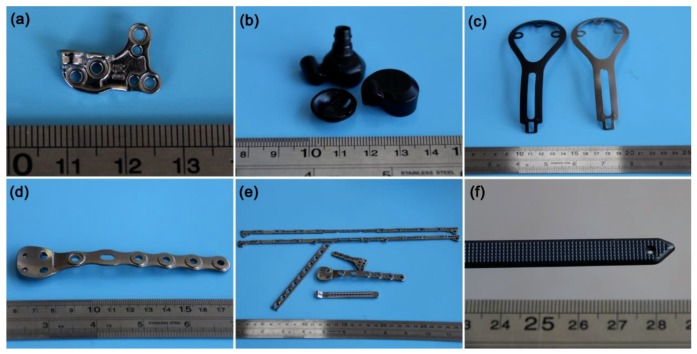
BMG parts with various shapes and used in different fields: (**a**) BMG transmission used in a notebook computer fabricated by Vit106; (**b**) thin-walled and hollow BMG earphones (Vit106); (**c**) Vit 106 thin-walled BMG samples coated in different colors; (**d–f**) biomedical implants fabricated using Vit105 BMGs.

Then, various Zr-based BMG parts were manufactured using the above glass formers and the EPV-HPDC process, as shown in Figure 7. The maximum thicknesses of the parts here is smaller than the corresponding critical size of glass formers, in order to ensure an amorphous structure. Figure 7a displays a transmission with lots of location holes. In the past, because of the shrinkage of liquid metal during solidification, a small part with lots of holes requiring a high center position accuracy and dimensional accuracy is extremely hard or even impossible to be formed by the casting process, and thus usually needs secondary processing, such as CNC. Our results show that the small shrinkage of the BMG and high solidification pressure in the EPV-HPDC can ensure that this type of part will be formed by just directly casting. In addition, it is well known that the thin-walled BMGs parts with a hollow structure are hard to form using the existing casting process, even using glass formers with an ultrahigh glass forming ability, such as Vit 1 containing toxic beryllium [4]. By using the strategies of filling the die in milliseconds and of solidification under high pressure, some hollow and thin-walled BMG parts, such as the earphone, are fabricated precisely using the EPV-HPDC process, and are displayed in Figure 7b,c. With regard to implant materials, the most common metals used are stainless steels, Co-Cr alloys, and titanium alloys [32]. BMGs are considered as a promising alternative type of material because of their outstanding mechanical properties, and corrosion and wear resistance [32]. Previously, because of the lack of corresponding molding methods, the excellent properties of BMG were limited in biomedical applications. Figure 7d–f displays some images of the amorphous implants shaped by the EPV-HPDC process. Some cases with a length dimension of 270 mm and with some fine embossments were formed successfully in one step, as shown in Figure 7e,f. According to the previous research, the cellular response to an implant intensively depends on its surface topography [33,34]. The ease in fabrication of the controlled topographical features on BMGs provides a practical possibility to functionalize the surfaces using the directly casting method (Figure 7f), rather than secondary processing like imprinting and microfabrication.

The production efficiency and part-cost economy are critical when a part is applied to engineering in a large scale. For efficiency, the EPV-HPDC process enables most of the near-net shaped BMG parts to be accomplished within 90 s, which is several times shorter than that using the existing method, such as thermoplastic forming [35] and the additive manufacturing process [36,37], and is comparable to that of the injection molding of BMGs. With regard to the economy, indeed, the raw material cost of BMGs is higher than that of the conventional materials, such as stainless steel, even though industrial-grade materials were used here. After considering the machining cost, however, the cost of BMG parts is competitive compared with that of a finished product manufactured using conventional materials and their associated processing methods. Taking the BMG smartphone frame as a sample, it was usually manufactured using the computer numerical control (CNC) machining of stainless steel in order to gain a high dimensional accuracy, high strength, excellent scratch resistance, and corrosion resistance. By this approach, tens of steps, including forging, heat treatment, machining, polishing, and so on, are needed. Consequently, the processing of these parts is time-consuming and costly. By using the EPV-HPDC process, it is found that equal or even higher levels of performance can be gained just by using one-step molding, and subsequently, a polishing treatment. On account of the high accuracy, efficiency, and economy of EPV-HPDC process, most of the above BMGs parts, such as the smartphone frame (Figure 5), transmission (Figure 7a), and earphones (Figure 7f), are being mass-produced in DongGuan Eontec Co., Ltd. (Dongguan, China). Our results show that the performance of the product is extremely stable (finished product ratio > 90%), demonstrating that the layout of the vertical and hot crucible in the present EVP-HPDC is reasonable and feasible for the industrial production of BMGs. 

Finally, it should be emphasized that the heat conductivity of the used steel mold (~33 W/mK) is only one-seventh of the copper mold (~230 W/mK) in suction casting [22], and an industrial purified raw material was used in the present work. With further improvement on the performances of the equipment, together with using a high purity material, the EPV-HPDC process can be used to fabricate BMGs with a wide range of compositions. As a vacuum forming method, EPV-HPDC offers a novel pathway for manufacturing other advanced materials, such as metallic glass matrix composites, high-entropy alloys, and nanostructured materials, in complex shapes. Furthermore, the flowing speed of the supercooled liquid and solidification pressure in the EPV-HPDC process can reach tens of meters per second and hundreds of megapascal [16], which are orders of magnitude larger than that of the common fabricating way of the BMGs, such as suction casting. Hence, the EPV-HPDC process provides access to study some of the critical scientific issues, such as the extreme fluid dynamics of glass forming liquid, shear-induced structural changes in fast-relaxing atomic liquids and melt [38,39], and relaxation kinetics of supercooled glass forming liquid under pressure [40], which are fundamental concerns for amorphous materials. As the EPV-HPDC equipment can be modified by standardized HPDC, the present approach is able to be implemented or reproduced easier than that of the previous reported routes [11,17,18]. Foreseeably, the EPV-HPDC process would have a huge impact on the discipline of amorphous alloys, as well as on our daily life.

## 4. Conclusions

In the present work, a novel but more common forming approach, named the entire process vacuum high pressure die casting (EPV-HPDC), was developed to prepare Zr-based BMGs possessing a higher melting point and chemical activity. The results indicate that the EPV-HPDC process can be used to produce a glassy structure for most of the common Zr-based bulk glass formers, even for marginal bulk glass formers from industrial grade raw material without a deterioration of strength. Various complex, especially thin-walled shaped Zr-based BMG parts, that are hard to be near-net shaped using the existing technologies, are formed by the EPV-HPDC process. It has been found that the novel forming approach has huge advantages over the dimensional accuracy of the as-cast parts, production efficiency, and product cost, compared with the other existing approaches. The development of the EPV-HPDC process paves the way for the large-scale industrial production and application of Zr-based BMGs, as well as other advanced materials.

## Supplementary Materials

The following is available online at http://www.mdpi.com/1996-1944/11/11/2338/s1. Video S1: Operating mode of EPV-HPDC.
